# Information Needs and Preferences of Men With Breast Cancer: A Qualitative Analysis of Internet Forum Posts

**DOI:** 10.1155/tbj/8821629

**Published:** 2026-01-07

**Authors:** Nicole Schemmel, Julia Lauberger, Julia Lühnen, Anke Steckelberg

**Affiliations:** ^1^ Institute of Health, Midwifery and Nursing Science, Medical Faculty of Martin Luther University Halle-Wittenberg, Martin Luther University Halle-Wittenberg, Halle (Saale), Germany, uni-halle.de; ^2^ Institute of Clinical Nursing Science, Charité–Universitätsmedizin Berlin, Freie Universität Berlin and Humboldt Universität zu Berlin, Berlin, Germany, charite.de

**Keywords:** breast cancer, health information, information needs, male breast cancer, patient information

## Abstract

**Background and Aims:**

Sex‐/gender‐specific health information for men with breast cancer is lacking. Health information supports patients in shared decision‐making. When developing evidence‐based health information, it is important to identify the patients’ information needs and preferences with regard to age, sex or gender, and other diversity aspects, including how the content is provided for the target group. However, studies show that sex/gender differences have rarely been considered. Our study investigates the information needs and preferences of cisgender men with breast cancer.

**Methods:**

A content‐structuring, qualitative content analysis of forum posts was performed. Internet forums and posts were selected according to the following criteria: relevance of the topic, English or German language, and public availability without registration. A qualitative content analysis according to Kuckartz was conducted. The selected posts were coded using MAXQDA.

**Results:**

A total of 1025 posts from three Internet forums were screened, and 96 posts were included for analysis—most of them from a German Internet forum. We identified seven main categories and 26 subcategories. Information needs and preferences are represented by the following main categories: “Epidemiology and general questions about the disease,” “Diagnostics,” “Therapy,” “Physician specialist services,” “Rehabilitation and lifestyle adaption,” and “Mental health.” Additionally, the “Preference for and access to current information” plays a role for the patients.

**Conclusions:**

Our study provides new insights into the information needs and preferences of men with breast cancer, mainly from German‐speaking countries. Providing accurate and reliable health information that meets patients’ needs and preferences is an ethical duty and has to be provided by healthcare systems. Such patient‐centered and inclusive health care will empower patients to make informed decisions.

## 1. Introduction

Cancer patients benefit from health information that meets their information needs and helps overcome information barriers. Studies have shown a correlation between information provision and health‐related quality of life (HRQoL), resulting in a better outcome in terms of HRQoL, as well as reduced anxiety and depression [1, 2]. In a study by Blödt et al. [3], cancer patients reported that information helped them to make crucial decisions they felt confident about. Participation in the decision‐making process allowed patients to take responsibility, leading to a sense of empowerment [3]. Therefore, assessing patients’ information needs and preferences and developing targeted health information appear beneficial. In the study by Iredale et al. [4], over half of the male breast cancer (MBC) patients reported a desire for gender‐specific information. The desire for and lack of sex‐/gender‐specific information was evident in various studies on MBC patients, according to the systematic review from Abboah‐Offei et al. [5]. When developing evidence‐based health information (EBHI) that offers relevant information and emotional support, it is essential to identify the information needs of the patients while considering age and sex differences and aspects of disability, and to tailor the content and design to the target group [6], as sex/gender differences result in differential health risks and health service needs, among other things [7]. The involvement of patients in the development process of EBHI ensures that patients’ needs and preferences are met [6]. This is what this study builds upon. The study aims to address the information needs and preferences of patients with breast cancer who were assigned male at birth. MBC is a rare condition, constituting less than 1% of all breast cancer cases and less than 1% of all male cancer cases. However, the incidence of MBC is on the rise [8, 9]. The most significant risk factor for men is the genetic predisposition, a major contributor being the breast cancer 2 (BRCA2) mutation [8, 9].

The systematic review by Chen and Wang [10] shows that the public commonly uses social media for seeking and sharing health information. People with health issues can benefit from online communities by giving and receiving information (e.g., about treatment options), experiences, and emotional support. In recent years, Internet forums have served as a data source for research [11], such as investigating patients’ experiences [10]. An Internet forum is a kind of virtual focus group where people can discuss and exchange information on specific topics. Their hierarchical structure makes it easy for researchers to navigate and locate relevant issues [11]. Analyzing online contributions enables researchers to access current thoughts, feelings, and needs, minimizing recall bias [11]. These forums often allow users to discuss more intimate or sensitive topics due to perceived anonymity [11, 12]. Despite these advantages, there are difficulties verifying information due to the anonymity of the Internet, and the interviewer cannot ask questions, so it may not be possible to collect details [11, 12]. However, the lack of interviewer influence on participant responses can be an advantage [11, 13]. Furthermore, Internet forums enable the inclusion of individuals from diverse geographical areas [13], which is an advantage when studying rare diseases, such as MBC.

In January 2023, we conducted a literature search to explore whether the topic “information needs and preferences of men with breast cancer” has already been studied using social media or forum analysis (for information about the literature search, see supporting information file 1). While no studies matching our criteria were found, Bootsma et al. [14] and Iredale et al. [4] investigated the topic using different methods. Bootsma et al. [14] conducted a mixed‐methods study in the Netherlands using focus groups and surveys involving men with breast cancer, their relatives, patient representatives, and healthcare professionals. The study identified unmet information needs related to symptoms, diagnosis, treatment, side effects, follow‐up, family and genetics, psychological impacts, coping strategies, and patient experiences. Iredale et al. [4] conducted a retrospective cross‐sectional study in the United Kingdom, using questionnaires to assess 161 men affected by breast cancer. The study found that 40.7% of respondents considered printed information less relevant and mainly targeted at women. The study also included in‐depth interviews that revealed desires for gender‐specific information and images of men in the information material. As the information needs and preferences of men with breast cancer have not been sufficiently investigated, this study aims to identify precisely what cisgender men with MBC require.

## 2. Methods

An interpretive hermeneutic design was chosen due to the exploratory nature of the objective [15]. This design involved conducting a content‐structuring, qualitative content analysis [16] of forum posts to reveal the information needs and preferences of men with breast cancer. The privacy of Internet forum users was a primary concern, and steps were taken to protect their data. The research project’s planning was aligned with the ethical guidelines of the Declaration of Helsinki [17] and local legal data protection regulations [18]. The study protocol received approval from the Ethics Committee of the Medical Faculty of Martin Luther University Halle‐Wittenberg (processing number 2022‐129). The work is guided by the Standards for Reporting Qualitative Research (SRQR) to ensure complete reporting and transparency and thus avoid reporting bias [19].

### 2.1. Access to the Research Field and Data Collection

Within this research, Internet forums are regarded as units of investigation, known as sampling units. For this study, these units were chosen from the entire pool of existing Internet forums using predetermined search terms and inclusion and exclusion criteria [15, 16]. An exploratory search was conducted (NS) in January 2023 via Microsoft Bing to detect forums related to MBC, using terms, such as “breast cancer,” “men,” and “forum” and their German translations. Bing was chosen owing to its high result validity [20] and its tendency to yield more precise results with multiterm queries than Google [21]. The websites displayed in the Bing search list were reviewed starting from the top search result. Inclusion criteria were as follows: Internet forums had to be thematically related to cisgender men with breast cancer or at least contain posts on the topic that could be filtered through a subforum or search function, such as a search bar or filtered picklists. Furthermore, the forums had to be conducted in either German or English. Forums requiring registration to access and read forum posts were excluded. Such forums are considered protected spaces for forum members [11], so we wanted to preserve their privacy for ethical reasons. Given the expectation of irrelevant or redundant search results and because it was not our goal to identify all existing forums on the topic, only the first 10 pages of search results were screened. Based on the inclusion criteria, Internet forums that seemed to be suitable were documented in a table and saved in the favorites bar of Microsoft Edge for easy retrieval.

The data collection took place between January and March 2023 (NS). To be included in the analysis, forum posts needed to meet the following criteria: relevance of post content to the topic of men with breast cancer, written in German or English. Initial posts and comments containing relevant questions or introducing new issues were considered. Excluded were posts where the need for information did not arise during initial reading. For instance, this was applied to photo posts, pure experience reports about healthcare providers or the sharing of Internet links. Posts in which individuals explicitly self‐identified as transgender were also excluded, as this population was not the focus of the study.

Owing to the abundance of data material on the Internet [16], it is recommended to limit the selection to a specific range. Therefore, it was planned to initially collect 25 posts from at least two different Internet forums and to select further posts in an iterative approach: The aim was to collect 10 more posts after each coding run until the category system no longer needed to be adjusted and data saturation could be assumed. During the data collection, relevant posts from the Internet forums were selected, starting from the first to the last subforum (top to bottom). Posts were chosen from each subforum, beginning with the most recent ones. Selected posts were copied into a Word document, and contributors were promptly anonymized. Data anonymization was achieved by erasing personally identifiable information from forum posts after collection [11]. Placeholder markers (^∗∗∗^) were used in their place. Unnecessary details, such as usernames and location, were omitted. The Word document was then imported into MAXQDA [22].

### 2.2. Data Analysis

The data analysis was based on content‐structuring, qualitative content analysis as described by Kuckartz and Rädiker [16], according to which the data were organized into main and subcategories, forming thematic categories reflecting major and minor themes. The analytical method is widely used for the content analysis of various types of data [23, 24].

The initiating phase involved marking relevant text segments in the forum posts and noting preliminary analysis ideas as memos. The second phase was about developing the main categories. For this study, an inductive approach was chosen to minimize confirmation bias. First, a test coding run was conducted, where around 17% of posts were initially coded. This was undertaken independently by two researchers (NS and JLa). Definitions for these main categories were promptly made. The two researchers then discussed the codes until a common consensus was reached. This was intended to make the codes assigned to the text passages and the category descriptions more reliable. With the developed preliminary category system, the first entire coding run of the data material was performed in the third phase (NS). This led to the inductive forming of new categories where text segments did not match the preliminary category system. In the fourth phase, the main categories were further differentiated into subcategories as texts were reviewed (NS). MAXQDA’s “List of Coded Segments” function facilitated this inductive process. Definitions for the subcategories were made. The fifth phase involved a second coding run with the established subcategories. The finalized category system was discussed (NS and JLa), and adjustments were made. When this category system had been established, the selected forum posts from the second data collection were coded (NS), revealing no need for further adjustments. Subcategory summaries were created using MAXQDA’s “Summary Grid” function. This flowed into the sixth phase, comprising a category‐based analysis oriented toward the main categories to explore what is explicitly or implicitly said about a particular topic. The seventh and final phase focused on documenting and presenting results in writing (NS), whereby prior work, such as summaries and memo contents, was integrated into the final text. The resulting report follows the main categories with anchor examples, promoting intersubjectivity.

With a view to publishing the study results, the German posts were translated into English with a back‐and‐forth translation after analysis. The forward translation was performed by the first author (NS) and the backward translation by an independent researcher (MA). A project memo was kept throughout the research project to document and report in detail on the methodological procedure and reflect on one’s research activity.

## 3. Results

### 3.1. Selection of Internet Forums and Posts

**Figure 1:**
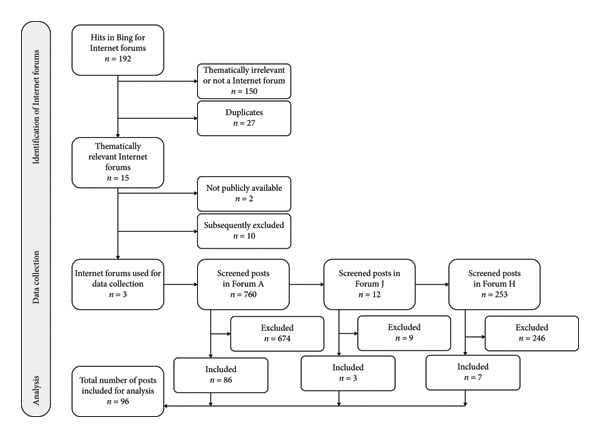
Flowchart of the data collection process.

The search for Internet forums on Microsoft Bing resulted in 163 hits with German search terms and 186 hits with English search terms. As only the search results (websites) from the first 10 pages were screened, this comprises 98 screened German hits and 94 screened English hits. After screening, 15 potentially relevant Internet forums were identified. The forums were labeled alphabetically from A to O. Two forums had to be excluded later due to the predefined criteria. One forum, an Internet forum from Germany, was directly related to the topic of men with breast cancer and was publicly available without registration (Forum A). No other forum with these two characteristics was identified. Consequently, Forum A was the primary source for selecting forum posts for the first data collection. In this initial data collection, after a screening of 760 forum posts, 90 posts were selected. Four posts were excluded after the first complete coding cycle due to the contributor’s unclear intention (question or moderation). For the second data collection, 10 additional posts were gathered from two Internet forums in the English language: Forum H from the United Kingdom had 12 posts related to MBC in the specific subforum at the time of data collection, all of which were screened. Three of these were included in the analysis. From the MBC subforum of the US‐based Forum J, from the newest to the oldest post, seven additional posts were selected for analysis after screening a total of 253 posts. A visual presentation of the data collection process is provided in the flowchart (Figure 1). For information about the characteristics of the Internet forums, see supporting information File 2. After the second coding process, the analysis revealed that the category system no longer needed to be adjusted. No additional posts were collected. Accordingly, the category system mainly reflects the information needs and preferences of cisgender men from Germany and German‐speaking countries.

### 3.2. Categories

**Figure 2:**
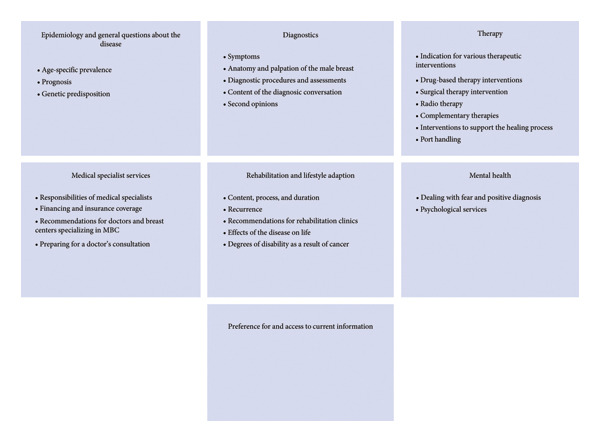
Overview of main categories and subcategories.

The content‐structuring content analysis on forum posts, guided by the research question “What information needs and preferences do men with breast cancer have?” yielded a category system comprising seven main categories. Most main categories were accompanied by a variable number of subcategories, totaling 26. The thematic contents of these categories are presented in the text below, and an overview of the main categories and subcategories is presented in Figure 2.

#### 3.2.1. Epidemiology and General Questions About the Disease

Within this main category, the need for basic disease‐related information becomes apparent. It encompasses three subcategories, each addressing specific aspects of the disease’s nature, such as its age‐specific prevalence within the population, its prognosis, and themes about genetic predisposition, such as facts about health risks and experiences regarding the use of preventive interventions, such as preventive mastectomy.

*“[…] at what age is a man at risk of developing breast cancer? Is it possible to narrow this down statistically?” (B018)*

*“[…] I wanted to ask what the chances of recovery are if a man is diagnosed with breast cancer.” (B051)*

*“[…] My treating gyn suggested a genetic consultation: i.e. blood test. If hereditary, then preventive surgery on other breasts. […] Does anyone have experience with this? Would be grateful for information.” (B047)*

#### 3.2.2. Diagnostics

The main category “Diagnostics” bundles the need for information on diagnostic criteria for disease detection, diagnostic procedures, and content of the diagnostic communication. The subcategory “Symptoms” summarizes the need for information about criteria that medical laypersons can also identify as an initial indication of the presence of the disease.

Further, the contributors asked for knowledge about the anatomy and palpation of the male breast.

*“[…] With such a hardening in this area, is breast cancer even to be considered? Or where exactly is the man’s “breast” located? […]” (B033)*

*“[…] I have searched the Internet in vain to find a procedure for palpating the breast. Can anyone give me tips on how to recognize a lump?” (B046)*

Also, the need for information on the indications, procedures, benefits, and risks of specific diagnostic procedures and assessments for breast cancer was expressed.

*“[…] I am afraid of this biopsy because I have learned […] that when the tumor is punctured, many tumor cells escape from the puncture site and spread throughout the body. Wouldn’t it be better to surgically remove the lump in any case and examine it pathologically and then decide on the next steps?” (B001K2)*

*“[…] is an MRI so conclusive that you can be sure that they [lymph nodes] are not malignant? […]” (B031)*

The contributors were interested in what information could be expected about the tumor (e.g., size and pathogenesis) in a diagnostic conversation, when using specific diagnostic procedures. There were also questions about understanding the meaning of medical terms in the context of diagnostics.

*“[…] What information about the tumor can I expect? Will I only be told that it is malignant or benign? Or does the pathologist determine the overall characteristics of the nodes. […]” (B009)*

*“[…] I also wonder what is meant by “space-occupying” object? Tumor? Cancer? Cyst? You cannot find a direct answer to this on the Internet.” (B045K1)*

If the contributors felt unsure about a doctor’s diagnosis, they asked for options for second opinions.

*“[…] Do I have to have another biopsy when I want to get a 2nd opinion from another breast center. […]” (B002K1)*

#### 3.2.3. Therapy

The main category “Therapy” combines information needs on indications, contraindications, implementation, risks, side effects, and process of various therapeutic interventions. Those seeking information asked for indications for different therapeutic interventions.

*“[…] Since the sentinel lymph node(s) are affected, the armpit was cleared out in a second operation. […] Do you get a stronger or longer chemo compared to the situation if everything was ok there?” (B045K5)*

The subcategory “Drug‐based therapy interventions” reflects the need for general information on various drug‐based interventions. The questions asked were about differences between the medications, indications regarding their use, application (type, process, and duration of medication intake), benefits, and potential short‐term and long‐term side effects, such as effects on sexuality.

*“What is the difference between Letrozole and Tamoxifen? […]” (B017K1)*

*“[…] Is a chemo like this undertaken on an outpatient setting, and do you get a port for it? Or in a hospital?” (B045K4)*

*“[…] I have been taking tamoxifen since the end of 2016, and I have been doing well with it […]. How long should I keep taking it? […]” (B028)*

*“[…] Since my partner is not thrilled about the side effects of Zoladex (quote from him: “I would rather die than not have an erection for 2 years”) […] We are both struggling with how he should decide. […] How do you deal with sexuality? […]” (B058)*

*“[…] As some of you have probably already noticed, a man or woman does not get tamoxifen in the pharmacy! Has anyone already switched to something else, such as Letrozole? Or how does one deal with the situation? […]” (B054)*

The subcategory “Surgical therapy interventions” presents the information needed about surgical therapy procedures regarding the organization and process of the interventions. In particular, those affected wanted to know when they could start doing certain activities again: How quickly would they be able to perform their everyday activities. They also wanted to know about the length of the hospital stay.

*“[…] How quickly did you get over the operation and its after-effects? How many days after the operation were you able to move normally again? Carrying things, jogging and cooking? […]” (B002)*

Some contributors asked for information about benefits, risks, and side effects of radiation therapy when weighing up the treatment options. Specifically, they asked about experiences concerning how the therapy affected the well‐being of a person.

*“[…] My question is, what are the advantages and disadvantages of radiotherapy? […]” (B013K1)*

*“[…] To what extent does radiation affect well-being? […]” (B053K4)*

Some contributors wish for reports of experiences about the benefits of complementary therapies, such as cannabidiol (CBD) drops or Hilo therapy (a form of cold therapy).

*“[…] the woman also had breast cancer. She now takes CBD drops, among other things. Does anyone have experience with this? […]” (B064)*

*“[…] Do any of you have experience with a cooling cap or Hilo therapy during chemo? […]” (B006K1)*

It was shown that patients need to be informed about the opportunities for their own participation and self‐performed interventions to support the healing process and avoid complications.

*“[…] To begin with, I would be interested in […] what you can do for the lymphatic drainage after the operation. […]” (B003)*

Those with a port system need information on handling a port and its temporal remain.

*“[…] Do you have already experience with a port? Is there anything to consider? […]” (B003K2)*

*“[…] does anyone have a tip on when is the right time to remove the port […]?” (B019)*

#### 3.2.4. Medical Specialist Services

The fourth main category thematically represents the need for information about a man’s use of gynecological specialist services. Some contributors did not know who the correct medical contact for men with breast cancer was, which indicates a need for information about the responsibilities of medical specialists.

*“[…] Now I have discovered a small lump in the area of the left nipple. […] I then called my urologist because I did not know who the right contact person was. […]” (B035)*

Furthermore, there was a need for information about financing and insurance coverage regarding medical services, such as how health insurance companies cover the costs of gynecological specialist services and examinations that male patients need.

*“[…] I received a referral for a breast MRI. A radiology practice told me that breast MRIs are only paid for in women and that I had to submit a cost estimate to the health insurance company. Have any of you had this kind of problem when you received a referral for a breast MRI during aftercare? […]” (B026)*

Some contributors asked for recommendations about specialists or breast centers. It was also interesting to see whether there were doctors with experience or who specialized in MBC.

*“[…] are there special clinics that treat breast cancer in men? […]” (B014)*

Additionally, tips on preparing for a consultation with a doctor were requested, especially advice on which topics should be addressed.

*“[…] I have an appointment at the breast center on Monday. Any tips on what I should/could think about or ask about? […]” (B005)*

#### 3.2.5. Rehabilitation and Lifestyle Adaption

The fifth main category bundles the need for information about follow‐up examinations, treatments, and programs, and the risk and cause of a recurrence, particularly regarding their lifestyle choices for prevention and early detection options for recurrences.

*“[…] Can someone tell me how their breast cancer aftercare for early-stage breast cancer […] works? […] What is done and how does the procedure at a doctor’s appointment for aftercare work? […]” (B022)*

*“[…] Please tell me, how do you do it with alcohol? […] But now I have been abstinent since the diagnosis (about 1.5 years). Before that, I did not drink much. Now, I do not drink alcohol at all for fear of a recurrence. How do you do it? How do you deal with that? […]” (B025)*

Additionally, there was a wish for recommendations regarding rehabilitation clinics and interventions. The need for information about which sex‐/gender‐specific rehabilitation interventions are offered appeared.

*“[…] Who has experience with the “special” arrival dates for men with breast cancer […] I have always felt very comfortable as a “middle-aged” man among all the ladies-but maybe now is the time for a new impulse. […]” (B061)*

For those affected, cancer is a formative turning point in life that involves questions about their future and where information about the long‐term effects of cancer on private and professional life is needed.

*“[…] I have never really been sick so far. Then suddenly, I am a cancer patient? Forever? I have so many questions in my head. […] Are you all still working, and are you just as resilient as you were before breast cancer? Are there perhaps even people here who have become self-employed despite cancer or who still run their own company? To what extent has cancer changed your life? […]” (B002)*

In addition, questions emerged regarding a possible degree of disability due to cancer and how to apply for this. The contributors also wanted to know what level they could expect and under what conditions a new application could be submitted.

*“[…] To what degree of disability were you assigned to? […] Does anyone have experience submitting a new application after 5 years? Does that make sense? As I have residual symptoms from the chemo […]” (B065)*

#### 3.2.6. Mental Health

The sixth main category deals with the need for information on how to deal with psychological and emotional stress, such as sleep disorders and fears caused by the illness.

*“[…] Since then, I have had an ever-increasing fear of breast cancer. How can I deal with this fear and uncertainty so that I can continue to live normally? Owing to my feelings of anxiety, I currently also have considerable sleep disorders. How do you deal with this? […]” (B001)*

Based on the fears that have just been listed, it can be assumed that there is a need for counseling and information, for instance, about maintaining a professional life or about work breaks during and after cancer, including financial and social assistance. These information needs can be implicitly read from the following quote:

*“[…] I do not know what I am going to do for a living. […] I am scared. Fear of being pitied by others. Fear of “crashing” professionally […] Fear of becoming poor—because of cancer. […]” (B002)*

#### 3.2.7. Preference for and Access to Current Information

The last main category shows how access to information and information material is preferred. It becomes evident that there is a general desire for up‐to‐date information.

*“[…] I am looking for the PDF file <<Breast cancer patient guide to the AGO recommendations 2022>>. The file must be from the year 2022, because I already have the one from 2021. […]” (B020)*

## 4. Discussion

Our study identified the information needs and preferences of men with breast cancer, primarily from German‐speaking countries. In summary, information needs on epidemiology and general questions about the disease were revealed, as well as on diagnostics and therapy. Other topics included medical specialist services, rehabilitation, and lifestyle adaption. Furthermore, information regarding mental health was requested. Additionally, men showed a preference for the latest information on the topic.

As shown in the systematic review from Abboah‐Offei et al. [5], the need for gender‐specific information is a present topic among men with breast cancer. Epidemiological and general questions, such as individual risk assessment and disease prognosis, raised in the forum posts, have previously been described in the literature [14]. Another aspect was the need for information on genetic predisposition. Men’s interest in understanding the implications of a positive genetic mutation diagnosis, for example, regarding preventive options and familial impact, has been found in studies from Strømsvik et al. [25] and Bootsma et al. [14]. Further, emotional distress and challenges in communicating genetic risks within families have been documented [25]. It can be concluded that information about the possible impact of genetic disposition on life and preventive interventions and counseling services should be available. Patients are also interested in how the topic can be discussed within the family.

Another finding of this study is the need for information about the process of diagnostic procedures. Questions about the symptoms were concise and consistent with other study results [14]. Additionally, the need for information on the anatomy of the male breast and its palpation as part of the self‐care examination played an essential role for the contributors for early detection of recurrence.

Questions were also asked about aspects of various therapeutic interventions, especially potential side effects [14, 26], such as sexual dysfunctions (e.g., loss of libido and erectile dysfunction). This is in line with the findings of other studies [14, 26, 27]. Some forum posts imply that potential sexual dysfunction as a side effect of treatment could influence the decisions for or against a therapy option. Furthermore, it reinforces the need to include in the patient guidelines any possible side effects affecting male bodily functions. Additional information needs involve complementary therapies, such as CBD drops, Hilo therapy, and interventions to stimulate lymphatic drainage. Questions were also asked about the correct handling of port systems and for how long it will be necessary as well as personal lifestyle choices (e.g., drinking alcohol). It can be deduced that patients would like information on how to avoid complications, and how to support and accelerate their healing process so that they can return to everyday life in a timely manner. Incorporating these topics into patient decision aids or EBHI could enhance the patient group’s desired self‐efficacy.

The forum posts revealed uncertainty and a lack of knowledge about healthcare provider roles and insurance coverage, particularly regarding male access to gynecological services, such as mammography. Some contributors assumed that urologists were the correct contact persons and not gynecologists, as is the case in Germany. To find the right contact person and be able to access medical services as quickly as possible, patients should be informed about which physician is responsible for their concerns. It is already known that in the German healthcare system, the possibility of using these gynecologic services for men is regulated differently in the areas of the Association of Statutory Health Insurance Physicians [28]. Consequently, patients must be informed about existing gaps in the healthcare system and be provided with clear guidance on whom to contact for support and information.

During the analysis, requests for information on recommendations for gynecologists specialized in MBC, breast centers, and rehabilitation clinics became apparent. The reason for this was not only the difficulty in finding a gynecologist for men but, above all, the desire for more sex‐specific information. Some contributors mentioned the wish to exchange information with other patients of the same sex or someone who already has experience with this patient group. Such an exchange was already considered helpful by MBC patients in the study by Farrell et al. [26]. Information for patients ought to include a reference to special rehabilitation clinic programs for men.

According to Faller et al. [2], men with cancer are provided with less information on the topic of psychological support than women, but also fewer needs in this regard were reported. The study by Brain et al. [29] on the psychological distress of men affected by breast cancer found that men suffer from cancer‐specific anxiety with intrusive thoughts. Our study also shows that the topic of mental health, e.g., dealing with negative feelings, such as anxiety, does play a role in MBC patients. Anxiety and the need for information about the impact of the disease on life and mental health can be found in this and other studies [14, 30–32]. Providing information on psychooncological and psychosocial services and self‐help strategies is therefore essential.

The category “Preference for and access to up‐to‐date information” does not represent a direct need for information in terms of content. The same applies partly to the subcategory “Content of the diagnostic conversation.” However, they are a helpful addition and indicate what ought to be considered when providing information. The former shows that the most up‐to‐date information is desired and should be easy to access and find. The latter mentioned subcategory suggests that some information, even when presented in medical terms in a person’s native language, may not always be easily understood by nonmedical professionals. Therefore, medical terminology should be explained in greater detail when used.

A review of the literature on informational needs among women with breast cancer revealed several themes, including diagnosis and treatment, family impact, coping with anxiety, leisure activities, self‐directed recovery strategies, sexual health, financial and insurance matters, and communication with healthcare professionals [33]. Similar themes were identified in our study; however, sex‐specific differences were evident. Male participants, for instance, reported a greater need for clarity regarding responsible medical specialists. Moreover, while some treatment side effects are shared by both men and women, their manifestation and impact—particularly concerning sexual functionality—can differ significantly and thus require tailored information. Topics, such as contraception, body image, breast reconstruction, and prosthesis use, were relevant for women but largely absent in men’s statements. These findings underscore the importance of sex‐ and gender‐sensitive approaches in addressing informational needs in oncology care.

### 4.1. Methodological Approach

Including data material from the Internet in a research project offers uncomplicated access to the field of interest due to its location independence. Particularly in the case of rare diseases, it could be a way of gaining access to these hard‐to‐reach patient groups, making sampling easier. Although the formal anonymity of the contributors is an advantage for them, regarding the protected area it provides for discussing sensitive topics, it might be a disadvantage for researchers. Apart from the fact that collecting demographic data from Internet forum users was not part of the original study design, such data acquisition would have been highly challenging. Although some users voluntarily disclose information, such as their place of residence, age, or occupation/education in their profiles or posts, this information is not available for all users. Therefore, analyzing posts in Internet forums to collect demographic data has inherent limitations in this regard, as it does not provide a reliable overview and thus limits the intersectional generalizability [34] of the results. These particularities must be considered depending on the aim of the research work.

The planned methodological procedure deviated from the study protocol at one point: Owing to the abundance of data on the Internet, it is necessary to limit the selection, as mentioned by Kuckartz and Rädiker [16]. Therefore, it was planned to select 25 posts from at least two different forums initially expecting few posts because of the disease’s rarity. However, a publicly accessible forum dedicated specifically to men with breast cancer was found and mainly used for data collection in our present study. The recommendation of Holtz et al. [11] was followed when selecting the Internet forums to ensure that the desired target group and sufficiently relevant analysis material were available. This made the selection of thematically relevant forum posts more targeted. As a result, far more forum posts than planned could be screened and selected. The forums for this second data collection were selected based on thematic relevance (dedicated breast cancer forums or health forums with corresponding subforums were favored over forums on various health topics) and navigation options (integrated search bars in the forum). We believe this was a suitable method for finding thematically appropriate forum posts and organizing the work steps more economically. When collecting data from the Internet forums, only the posts deemed relevant after the initial reading (see inclusion and exclusion criteria in the method section) were collected for analysis, not entire discussion threads, to reduce data volume. Despite concerns about losing context, the posts remained complete and understandable, proving that this approach has been successful.

### 4.2. Strengths and Limitations

This study is a retrospective analysis using data created for a different purpose and was not explicitly generated for this research work. Thus, the research method did not allow for an exchange with the sample, as the interview method does [15]. Subsequently, there was no interaction between the researcher and the people being studied. Although this ensures that researchers cannot influence those being studied, they are also unable to ask questions to understand or validate statements. Only Internet forums or forum posts that used English or German were included in the analysis. Additionally, most of the selected posts came from a German‐speaking forum with an imprint from Germany, so the information needs and preferences of people from German‐speaking countries were primarily determined in this study. Structure, treatment pathways, and circumstances of the German healthcare system, for instance, are not applicable to those of other countries and thus may influence information needs and preferences. The inferential generalizability may not be given [34]. Consequently, the results are particularly relevant for information providers from German‐speaking countries.

In the design of this study, we focused on cisgender men. Accordingly, forums or subforums predominantly used by transgender persons were excluded, as were posts in which contributors explicitly stated that they do not identify as cisgender men. However, due to the anonymous nature of Internet forums, it is impossible to definitively verify the sex and gender identity of every individual contributor. Consequently, a degree of uncertainty regarding sex and gender of contributors remains. It is also important to note that the study included only men with breast cancer who use Internet forums. This group may differ from the broader population of males with breast cancer in terms of information‐seeking behavior and digital health literacy.

The timing of data extraction is somewhat in the past; however, it represents an important consideration for interpreting our findings. We hypothesize that the change of information needs is influenced by structural aspects of healthcare delivery—particularly the quality, availability, and dissemination of patient information [35, 36]—but that these structural factors evolve at a slower pace. We thereby imply that there is limited impact on the category system employed in this study. Nevertheless, we recognize that, e.g., because of novel developments in therapies, changes in information needs and preferences cannot be precluded.

Notably, this work was carried out as part of a university qualification, and the first author (NS), therefore, has less experience in scientific work. A female researcher who is more experienced in the methods used here (JLa) was hence included as a discussion partner while creating the category system. The first author has no direct or indirect connection to the topic of men with breast cancer or even contact with the patient group. Due to the female gender of the two researchers and possible distortions due to unconscious expectations, unsuitable coding or category descriptions may have arisen.

### 4.3. Implications for Practice and Further Research

In their qualitative synthesis of reports from men living with breast cancer, Quincey et al. [37] found that this disease is currently still attributed to the female sex in the Western world. Men with breast cancer have their self‐image and body image as men shaken by a “woman’s disease” [29–32]. However, there are divergent perceptions of masculinity within ethnicities and cultures, which, for example, influence interpersonal relationships, such as emotional closeness or distance [38]. We assume that this may also have an impact on how information should be provided in different cultural contexts, for instance. This raises the question of patients’ information needs and preferences in other ethnic groups and cultures and whether these differ from those reported here. This offers further research opportunities. Another point is that our study is dedicated to the information needs and preferences of cisgender men with breast cancer. The information needs and preferences of trans men or trans women were not assessed. However, Blok et al. [39] argued that the existing guidelines on breast cancer screening are also relevant for transgender people. We would like to add that it cannot be excluded that, apart from transsexuality, other information needs and preferences may arise due to other gender identities. Developers of health information should consider the diversity of needs and preferences of patient groups.

## 5. Conclusion

This research contributes to identifying the information needs and preferences of men with breast cancer, particularly of those from German‐speaking countries. This may guide the development process of patient decision aids and EBHI and thus improve health care for this patient group. Health information for patients is an important task that falls within the governments’ responsibilities. The government facilitates a patient‐centered and inclusive healthcare system that empowers patients to make informed decisions by ensuring access to accurate and reliable information that meets patients’ needs and preferences.

**Nomenclature**
BRCA: Breast cancer [gene]
CBD: Cannabidiol
EBHI: Evidence‐based health information
HRQoL: Health‐related quality of life
MAXQDA: [MAX] qualitative data analysis
MBC: Male breast cancer
MRI: Magnetic resonance imaging

## Disclosure

All authors read and approved the final manuscript. This funding source was not involved in the study design, data collection, analysis, interpretation of data, or the writing of this manuscript.

## Conflicts of Interest

The authors declare no conflicts of interest.

## Author Contributions

Julia Lühnen and Anke Steckelberg supervised the research. Nicole Schemmel conceptualized the study with methodological guidance and critical revision from Julia Lauberger, Julia Lühnen, and Anke Steckelberg. Nicole Schemmel collected all data and performed the data analysis. Julia Lauberger was involved in the test coding run, which included a discussion about the preliminary category system and the category system discussion. Nicole Schemmel first drafted the article. All the authors critically revised the article for intellectual content. Nicole Schemmel had full access to all of the data in this study and takes complete responsibility for the integrity of the data and the accuracy of the data analysis.

## Funding

We acknowledge the financial support of the Open Access Publication Fund of the Martin Luther University Halle‐Wittenberg for covering the fees associated with the open access publication. This research did not receive any other funding.

## Supporting Information

Additional supporting information can be found online in the Supporting Information section.

## Supporting information


**Supporting Information 1** File 1 contains information about the literature search, like time of conduction, used databases, search terms, inclusion, and exclusion criteria, and the search result.

**Supporting Information 2** File 2 contains information about the characteristics of included internet forums, such as language, number of registered members, and number of posts.

**Supporting Information 3** File 3 contains the Standards for Reporting Qualitative Research (SRQR) Checklist completed for this research.

## Data Availability

The data that support the findings of this study are available from the corresponding author upon reasonable request.
