# Supplementary material for: Information Needs and Preferences of Men With Breast Cancer: A Qualitative Analysis of Internet Forum Posts
Source: Breast J. 2026 Jan 7;2026:8821629. doi: 10.1155/tbj/8821629 (PMC12779508; doi:10.1155/tbj/8821629)
Supplement: Supplementary file 1 — Supporting Information 1 File 1 contains information about the literature search, like time of conduction, used databases, search terms, inclusion, and exclusion criteria, and the search result. [file TBJ-2026-8821629-s002.docx]

In January 2023, a literature search was conducted in the databases MEDLINE (via PubMed), CINAHL, and PSYNDEX (via EBSCOhost), to explore whether the topic `information needs of men with breast cancer` already has been studied using social media or forum analysis. Additionally, a snowball search was carried out by examining the reference lists of relevant publications to identify potentially relevant studies.

First of all, an orientating search was carried out in Pubmed to identify suitable search terms. Synonyms and translations were determined for these terms (see Table 1 below) and truncations were used. Search strings were then defined that were compatible with the databases. For this purpose, the words of each module were linked using the Boolean operator OR, and the modules were linked to each other using the Boolean operator AND.

The inclusion criteria were as follows: Publications in English or German, that discussed information needs, preferences, experiences, and perceptions of men diagnosed with breast cancer and utilized data collection from social media and online networks. Studies meeting these criteria in their abstracts were considered for full-text review. Since limited literature on the topic was expected and to avoid overlooking relevant publications, no additional exclusion criteria were initially set.

The search in MEDLINE yielded 647 hits. CINAHL yielded 166 hits and PSYNDEX three hits. After screening the abstracts and titles, two studies were found that met our criteria for population, disease and outcome, but not the research area we were interested in. In the end, no study met our complete list of criteria.

Table 1: Search terms

| **Population** | **Outcome** | **Disease** | **Field** |
| --- | --- | --- | --- |
| Mann  Männer  Man  Men  Male | Education needs  Preference  Präferenz  Experience  Erfahrung  Perspective  Decision  Entscheidung  Information sources  Information needs | Breast cancer  Mastocarcinoma  Mammakarzinom  Brustkrebs | Social media  Soziale medien  Social network  Soziale netzwerke  Online community  Internet forum  Online Forum  Online group  Post |
