# Supplementary material for: Information Needs and Preferences of Men With Breast Cancer: A Qualitative Analysis of Internet Forum Posts
Source: Breast J. 2026 Jan 7;2026:8821629. doi: 10.1155/tbj/8821629 (PMC12779508; doi:10.1155/tbj/8821629)
Supplement: Supplementary file 2 — Supporting Information 2 File 2 contains information about the characteristics of included internet forums, such as language, number of registered members, and number of posts. [file TBJ-2026-8821629-s003.docx]

| **Forum** | **Characteritics** | | | | |
| --- | --- | --- | --- | --- | --- |
|  | **Residence/ language** | **Number of registered members** | **Number of posts (incl. comments)** | **Content and structure** | **Other** |
| A | Germany, German | 102 | 760 | - Forum specialised in the topic of male breast cancer - Divided in seven subforums - posts are displayed starting with the most recent one | - No registration for access and reading posts is required - guests can also contribute posts without signing up |
| H | United Kingdom, English | 75,900 | 12 (subforum) | - Breast cancer forum that includes a subforum about men with breast cancer - subforum itself is not further categorized thematically - posts are displayed starting with the most recent one | - No registration for access and reading posts is required |
| J | United States of America,  English | not determinable | 2,255 (subforum) | - Breast cancer forum that includes a subforum about men with breast cancer | - No registration for access and reading posts is required |

Please note that these numbers and characteristics are based on the time of data collection as described in the paper.
